# Generation of New *Hairless* Alleles by Genomic Engineering at the *Hairless* Locus in *Drosophila melanogaster*

**DOI:** 10.1371/journal.pone.0140007

**Published:** 2015-10-08

**Authors:** Heiko Praxenthaler, Thomas K. Smylla, Anja C. Nagel, Anette Preiss, Dieter Maier

**Affiliations:** Universität Hohenheim, Institut für Genetik (240), Garbenstr. 30, 70599, Stuttgart, Germany; National Institutes of Health (NIH), UNITED STATES

## Abstract

Hairless (H) is the major antagonist within the Notch signalling pathway of *Drosophila melanogaster*. By binding to Suppressor of Hairless [Su(H)] and two co-repressors, H induces silencing of Notch target genes in the absence of Notch signals. We have applied genomic engineering to create several new *H* alleles. To this end the endogenous *H* locus was replaced with an attP site by homologous recombination, serving as a landing platform for subsequent site directed integration of different *H* constructs. This way we generated a complete *H* knock out allele *H* ^*attP*^, reintroduced a wild type *H* genomic and a cDNA-construct (*H* ^*gwt*^, *H* ^*cwt*^) as well as two constructs encoding H proteins defective of Su(H) binding (*H* ^*LD*^, *H* ^*iD*^). Phenotypes regarding viability, bristle and wing development were recorded, and the expression of Notch target genes *wingless* and *cut* was analysed in mutant wing discs or in mutant cell clones. Moreover, genetic interactions with *Notch* (*N* ^*5419*^) and *Delta* (*Dl* ^*B2*^) mutants were addressed. Overall, phenotypes were largely as expected: both *H* ^*LD*^ and *H* ^*iD*^ were similar to the *H* ^*attP*^ null allele, indicating that most of H activity requires the binding of Su(H). Both rescue constructs *H* ^*gwt*^ and *H* ^*cwt*^ were homozygous viable without phenotype. Unexpectedly, the hemizygous condition uncovered that they were not identical to the wild type allele: notably *H* ^*cwt*^ showed a markedly reduced activity, suggesting the presence of as yet unidentified regulatory or stabilizing elements in untranslated regions of the *H* gene. Interestingly, *H* ^*gwt*^ homozygous cells expressed higher levels of H protein, perhaps unravelling gene-by-environment interactions.

## Introduction

Communication amongst cell neighbours is made possible by the Notch signalling pathway, driving cell specification and differentiation [1–3]. In *Drosophila*, Notch signalling is activated by the binding of one of the membrane-tethered ligands Delta (Dl) or Serrate (Ser), present on one cell, to the Notch receptor present on the neighbouring cell. This interaction results in the cleavage of the Notch receptor and the release of its intracellular domain (ICN). ICN mediates the formation of an activator complex together with the DNA-binding protein Suppressor of Hairless [Su(H)] and the co-activator Mastermind (Mam), entailing the transcriptional activation of Notch target genes [4–6]. In the absence of Notch signalling activity, Notch target genes are silenced by a repressor complex consisting of Su(H) bound to Hairless (H) and the two co-repressors Groucho (Gro) and C-terminal binding protein (CtBP) [7–10].

Hairless (H) is the major antagonist of the Notch signalling pathway in *Drosophila*, negatively regulating the majority of Notch mediated events during imaginal development (overview in [11]). The role of H has been investigated in the past with classical mutations and with the overexpression of wild type and mutant H constructs. The latter has been used for a systematic structure-function analysis of the H protein and allowed the localization of the Su(H)-, the Groucho- and the CtBP-binding domains, respectively (SBD, GBD, CBD) [7,8,10,12–19]. Moreover, it was shown that the mutation of a single residue within the SBD (Leucine 235 to Aspartate) completely abolished the H-Su(H) interaction [15]. Tissue specific overexpression of H results in a plethora of phenotypes as a consequence of the downregulation of Notch activity [7,10,12–15,18–21]. Albeit very helpful for a detailed analysis of specific processes, the overexpression of H is quite dramatic and does not compare to an analysis of mutants expressed under wild type regulation. Therefore, we made use of the recently established method of genomic engineering in *Drosophila* [22] to replace the endogenous *H* locus with an attP site by homologous recombination. The resultant *H* ^*attP*^ founder line was genetically and molecularly verified as a complete *H* knock out allele. It further served as landing platform for the subsequent site-directed integration of various *H* constructs, two wild type and two mutants affecting Su(H) binding. A detailed characterization of these new *H* alleles is presented, furthering our insight into the structure and function of the *H* gene.

## Results

### Generation of the *Hairless* knock out line

**Fig 1:**
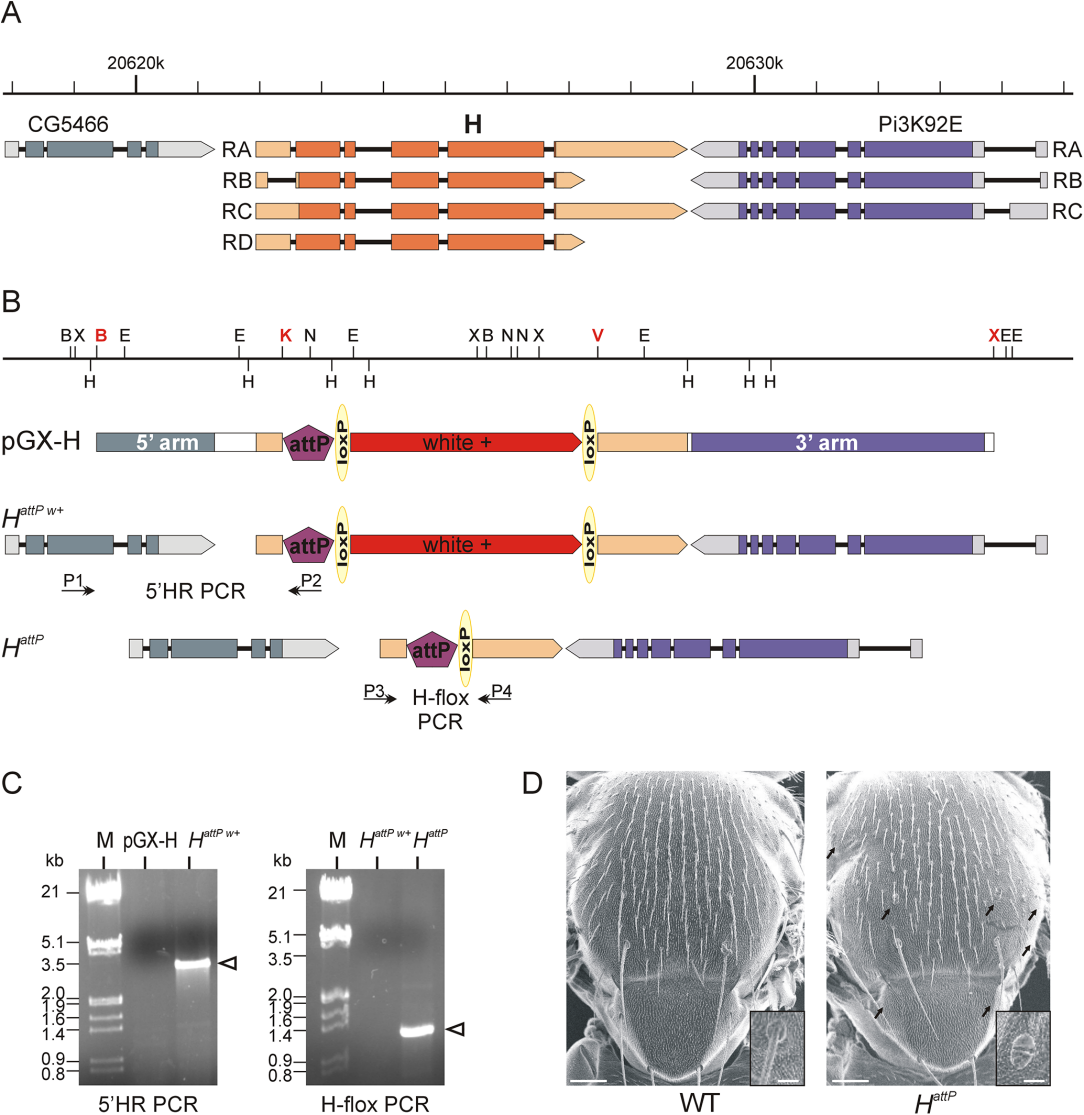
Genomic engineering at the *Hairless* locus. (A) The *Hairless (H)* locus is flanked by the *CG5466* and *Pi3K92E* genes. The scheme depicts the major transcripts; arrowheads point in the direction of transcription. The coding region is coloured, UTRs are shown paler (according to http://www.flybase.org, released 2014_06). (B) Restriction map of the genomic *H* wild type region including the following restriction sites: B, *Bam* HI; E, *Eco* RI; H, *Hin*d III; K, *Kpn* I; N, *Not* I; V, *Eco* RV; X, *Xho* I. For the engineering, a 3 kb *Bam* HI–*Kpn* I fragment was cloned as 5’arm and a 6.4kb *Eco* RV—*Xho* I fragment as 3’arm into pGX-attP to give pGX-H; respective restriction sites are in red. The vector contains a *white* ^+^ gene for selection, an attP site for site specific integration and two loxP sites for marker removal. The respective elements are not to scale. Homologous recombination at the *H* locus results in the founder knock out line (*H* ^*attP w+*^). Excision of *white* ^+^ by CreI-recombinase results in the knock out line *H* ^*attP*^, lacking about 5.1 kb of the *H* locus, i.e. the entire coding region. Primer pairs used for genotyping are indicated; they are not to scale. (C) Fly lines were genotyped by PCR. Left panel: Homologous recombination at the *H* locus was confirmed with 5’HR PCR: primer P1 binds upstream of the 5’arm and primer P2 within attP site to give ~3.1 kb fragment in the *H* ^*attP w+*^ line, but not the starting line (pGX-H). Right panel: Excision of *white* ^*+*^ in *H* ^*attP*^ was confirmed with H-flox PCR: using primer pair P3/P4 flanking the deletion, a ~1.3 kb fragment is seen in *H* ^*attP*^ but not in the starting line *H* ^*attP w+*^. (M, marker λ-DNA, *Eco* RI/*Hin*d III digested; numbers are appr. size in kb). (D) Scanning electron micrographs of thoraces from wild type (WT) (left panel) in comparison to a heterozygous mutant *H* ^*attP*^ (right panel). *H* mutants are typified by loss of bristles; arrows point to examples of macrochaetae loss. The inset shows an enlargement of an orbital bristles; note the double socket in the mutant. Scale, 100 and 20 μm, respectively.

Genomic engineering according to Huang et al. [23] was employed to generate the *Hairless* knock out founder line *H* ^*attP*^ as outlined in Fig 1. To this end, genomic DNA fragments flanking the *Hairless* locus were cloned into pGX-attP and the resultant construct pGX-H was introduced by P-element mediated transformation into the fly genome (Fig 1A and 1B). Homologous recombination [24] generated the Hairless founder line *H* ^*attP w+*^ where a *white* ^+^ gene replaced the *Hairless (H)* gene (Fig 1B). Subsequent elimination of the *white* ^+^ marker by Cre-lox mediated recombination resulted in the final *Hairless* knock out line *H* ^*attP*^ containing the attP site and one loxP site in place of the original *H* locus (Fig 1B). The genotype was confirmed by PCR and sequence analysis (Fig 1C).

As *H* ^*attP*^ carries a complete deletion of the *H* coding sequence it is expected to be a complete null allele. Accordingly, *H* ^*attP*^ is a recessive lethal and displays the typical haplo-insufficient *H* phenotypes, i.e. loss of macro- and microchaetae, frequently accompanied by a transformation of bristle shaft into socket, causing a double-socket phenotype (Fig 1D) [25–27].

### Rescue of the *H* ^*attP*^ knock out line with wild type *H* DNA

**Fig 2:**
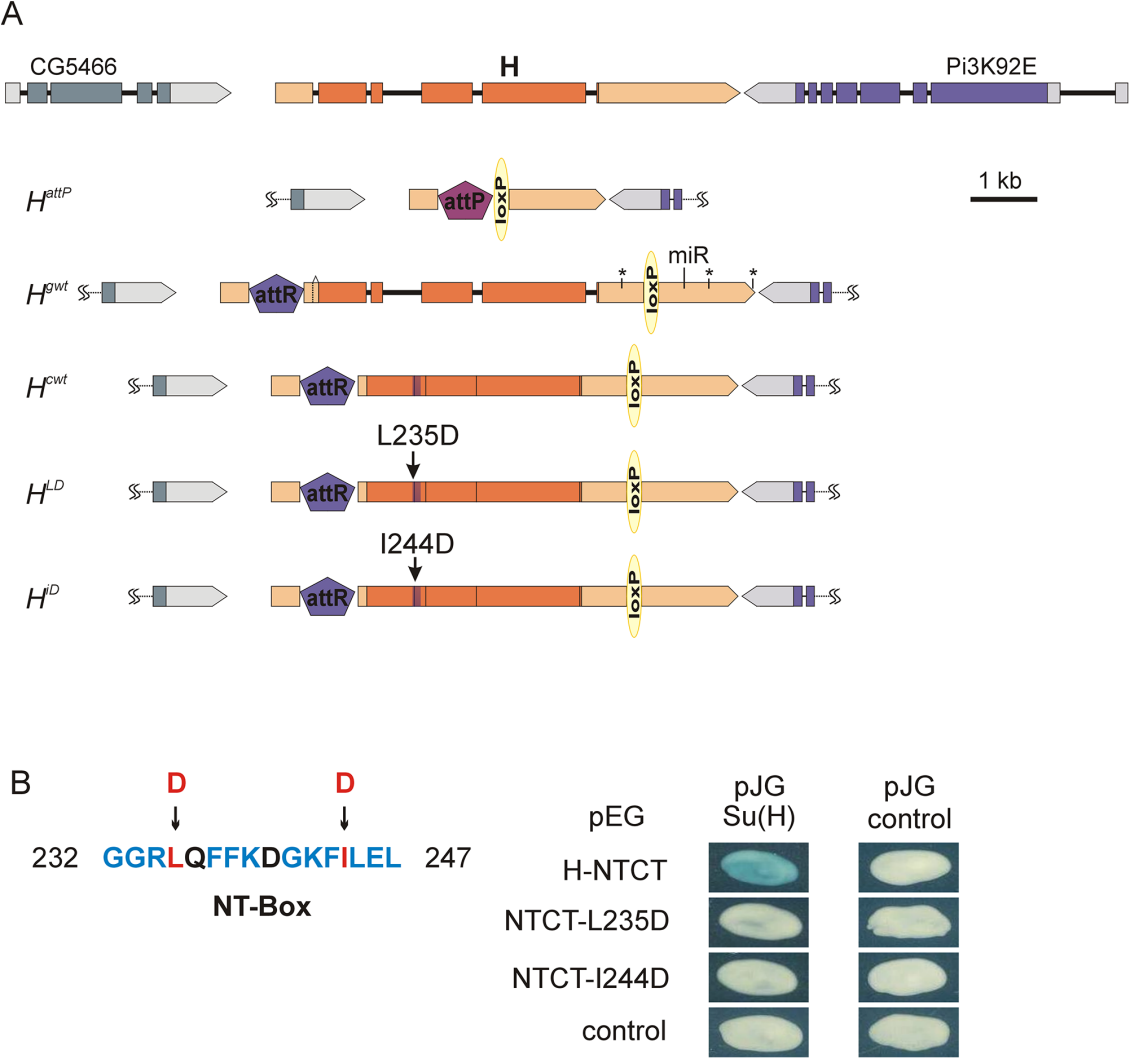
Newly generated *H* alleles. (A) Schematic representation of the newly generated *H* alleles after floxing (see also S1 Fig); the neighbouring loci are clipped. Colours and symbols are as in Fig 1. Only att- and loxP sites are not to scale (bar, 1 kb). *H* ^*attP*^ carries a 5.1 kb deletion, removing the entire coding region and some of the 5’ and 3’ UTR of *H*. *H* ^*gwt*^ differs from wild type only by the attR site plus residual vector sequences (total of 109 bp) in the 5’UTR and the loxP site plus residual vector sequences (total of 122 bp) in the 3’ UTR. A small intron in the 5’UTR is differentially spliced (dashed line). Presumptive polyadenylation sites (*) and the miR305-target (miR) in the 3’UTR of *H* are indicated. None is affected by the loxP site. *H* ^*cwt*^ carries cDNA instead of genomic DNA, i.e. is lacking the introns, however, carries a 136 bp duplication in the 3’UTR (S1 Fig). The Su(H) binding domain covers the first intron and is shaded purple. Position of point mutations in *H* ^*LD*^ (L235D) and *H* ^*iD*^ (I244D) is indicated. (B) Amino acid sequence of the NT-Box of H protein known to bind to Su(H) [15]. Conserved amino acids are highlighted in blue or red; red marks the mutated positions. Protein binding of H-NTCT wild type and mutant constructs (in pEG) to full length Su(H) (in pJG) was probed in a yeast two-hybrid assay. Empty vectors served as control. H-NTCT comprises amino acids 171–357 and overlaps the Su(H) binding domain [15,30]. In NTCT-L235D, Leucine at position 235 and in NTCT-I244D, Isoleucine at position 244 was each changed to Aspartate. The L235D mutation was shown before to destroy Su(H) binding [15]. Likewise does the I244D mutation.

**Fig 3:**
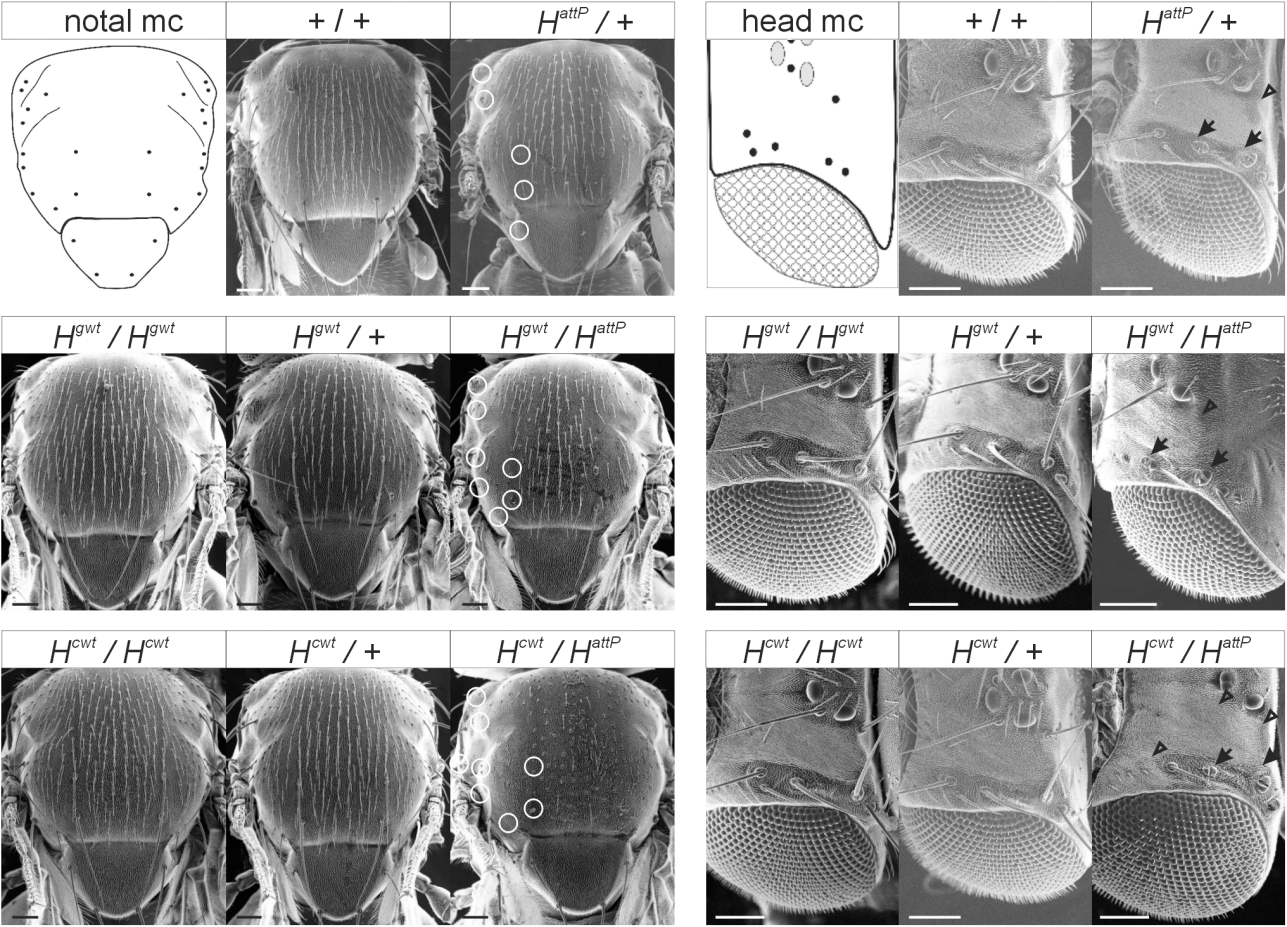
Rescue of *H* ^*attP*^ with wild type *H* sequences. Scanning electron micrographs of representative flies of the given genotype. *H* ^*gwt*^ and *H* ^*cwt*^ were either crossed to wild type Oregon R (+) or to *H* ^*attP*^ to allow for a comparison of homo-, hetero- and hemizygous flies. Oregon R served as control. 26 macrochaetae are present on the notum (notal mc) and 14 on the head (head mc) at fixed positions shown in the schemes. To highlight the phenotype, lacking macrochaetae are encircled on the left heminotum. Moreover, shaft to socket transformations on the head are marked with an arrow and loss of the complete bristle organ by an arrowhead. Note in addition the loss of many microchaetae on the notum of hemizygous *H* ^*cwt*^ */H* ^*attP*^ flies. Size bars, 100 μm.

We took advantage of the attP site within the *Hairless* locus to re-integrate wild type forms of the *H* gene with the help of PhiC31-integrase as outlined before in [28] (S1 Fig). We used genomic as well as *H* cDNA to generate *H* ^*gwt*^ and *H* ^*cwt*^ alleles, respectively (Fig 2A). Due to the construction, the attR side resides within the 5’, and the loxP site within the 3’ untranslated region (UTR) of the *H* gene. None of them affects known regulatory motifs like e.g. polyadenylation sites, the described binding site of miR–305 [29] or of other potential micro-RNA targets (Fig 2A and S1 Fig). Both genomic and cDNA were apparently fully functional: *H* ^*gwt*^ and *H* ^*cwt*^ homozygous stocks were viable and fertile and phenotypically indistinguishable from wild type indicating that both alleles allow normal fly development (Fig 3 and S2 Fig). As expected, the heterozygotes were indistinguishable from wild type (Fig 3). Moreover, the hemizygotes and the balanced heterozygous siblings hatched at similar rates (*H* ^*gwt*^/*H* ^*attP*^ 97.4% of *H* ^*gwt*^/TM6B [n = 995], and *H* ^*cwt*^/*H* ^*attP*^ 104.4% of *H* ^*cwt*^/TM6B [n = 967]). The hemizygous condition, however, uncovered a subtle to weak loss of activity of both rescue alleles: *H* ^*gwt*^/*H* ^*attP*^ flies developed statistically 20% less bristles on head and thorax compared to +/*H* ^*attP*^ flies (Fig 3 and S2 Fig), and *H* ^*cwt*^/*H* ^*attP*^ flies had just 50% of the bristles seen in the hemizygotes (Fig 3 and S2 Fig). As bristle development in *H* mutants is highly susceptible to genetic background (Nash 1969), the difference between wild type and *H* ^*gwt*^ may be negligible. Compared to *H* ^*gwt*^, however, *H* ^*cwt*^ apparently had reduced *H* activity, given that they were induced in the same genetic background (Fig 3 and S2 Fig). The only difference between *H* ^*gwt*^ and *H* ^*cwt*^ are the introns and for technical reasons, a small sequence duplication in the 3’UTR, raising the possibility of regulatory or stabilizing elements residing within these sequences (Fig 2A and S1 Fig).

### *H* mutants defective of Su(H) binding

**Fig 4:**
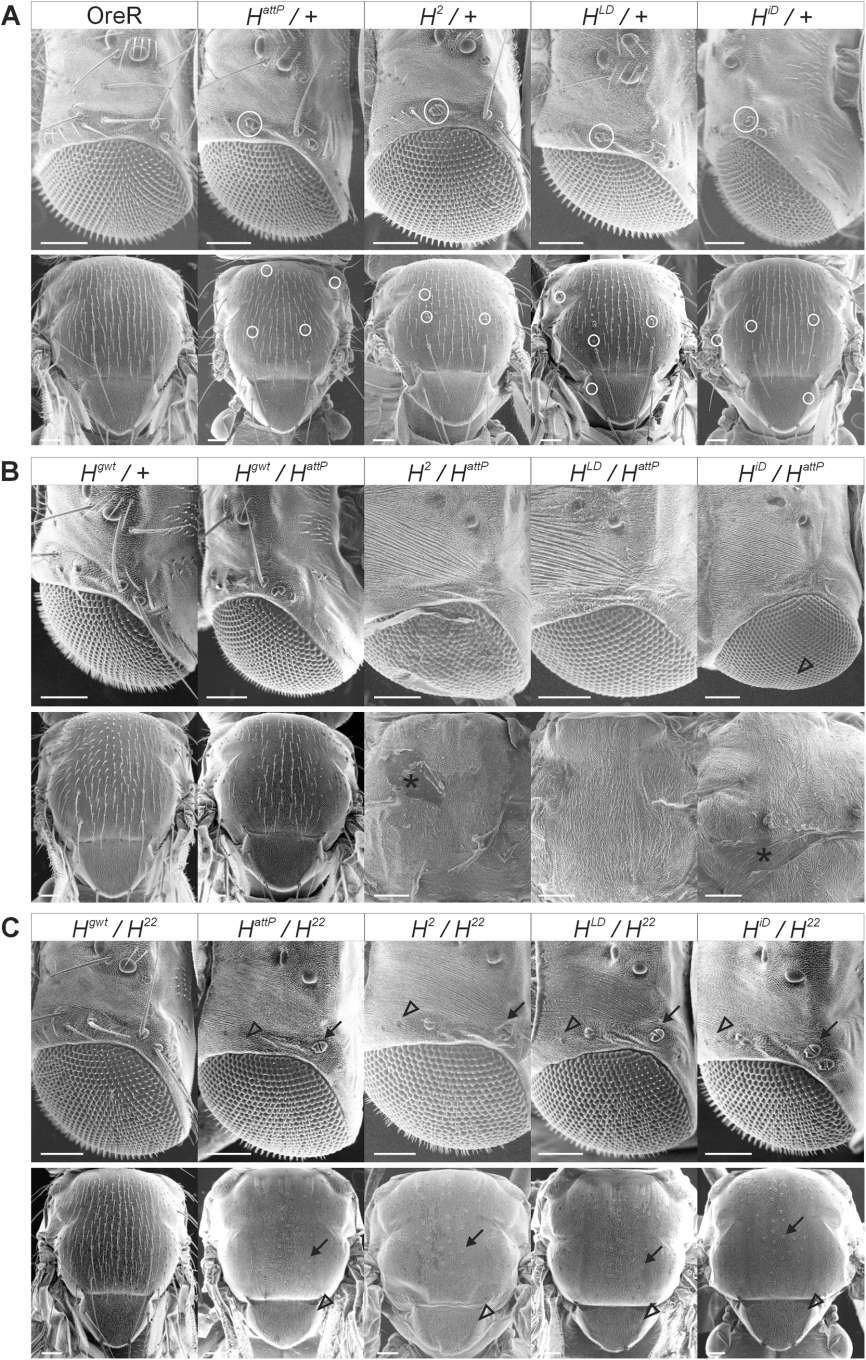
Phenotypic analysis of *H* mutant phenotypes. (A) Phenotypes of heterozygous flies of the given genotype; + represents the third chromosome of *y* ^*1*^ *w* ^*1118*^, except for *H* ^*attP*^ which is over wild type Oregon R (OreR). A few examples of bristle loss or shaft to socket transformation are encircled. (B) Hemizygous animals of the given genotype. Control is *H* ^*gwt*^/+ Oregon R and *H* ^*gwt*^/*H* ^*attP*^. Only a few of the hemizygous *H* ^*2*^, *H* ^*LD*^ and *H* ^*iD*^ animals developed to pharate adults that were dissected from the pupal case. They are completely bald, except for a few interommatidial bristles in *H* ^*iD*^ */H* ^*attP*^ (arrowhead points to an example). Asterisk marks example of remains of the pupal envelope. (C) Trans-heterozygous combination of the various *H* alleles with the weak allele *H* ^*22*^. Note the complete shaft to socket transformation of both macro- and microchaetae on head and thorax. Examples are highlighted by arrows. In addition, transformation of outer into inner cell fates results in a partial or complete disappearance of the bristle organs [25]; some examples are marked by arrowheads. Incomplete transformation is best seen on the head. Note that interommatidial bristles are largely unaffected. Size bars in (A-C), 100 μm.

The Su(H) binding domain of H contains two highly conserved boxes, the NT- and the CT-Box [30]. The contact to Su(H), however, requires only the NT-Box [15,30]. In a yeast two-hybrid assay using the overlapping NTCT construct, Leucine at position 235 was shown to be critical for the Su(H) contacts, since its mutation to Aspartate destroyed the H-Su(H) binding [15] (L235D; see also Fig 2B). A second point mutation was introduced in NTCT, replacing Isoleucine at position 244 with Aspartate (I244D; Fig 2A). A yeast two-hybrid experiment confirmed that the I244D mutation suppressed the binding of the H-NTCT domain to the full length Su(H) protein (Fig 2B) [15]. The mutants were introduced in the *H* cDNA and inserted into the *H* locus using the *H* ^*attP*^ founder as outlined above (S1 Fig and Fig 2A). The resultant mutant alleles, *H* ^*LD*^ and *H* ^*iD*^, were recessive lethal and lethal in trans over *H* ^*attP*^ as well as over the defined alleles *H* ^*2*^ and *H* ^*P8*^ [27,31]. Heterozygotes displayed the typical bristle loss (Fig 4A). In the hemizygous trans-combination over *H* ^*attP*^ pharate adults were obtained on rare occasions that lacked bristles altogether (Fig 4B). Overall, the phenotypes were very similar to the *H* ^*attP*^ and *H* ^*2*^ alleles [25] (Fig 4A and 4B), indicating that both *H* ^*LD*^ and *H* ^*iD*^ had lost most of H activity.

### Growth defects and protein expression in the *Hairless* mutants

**Fig 5:**
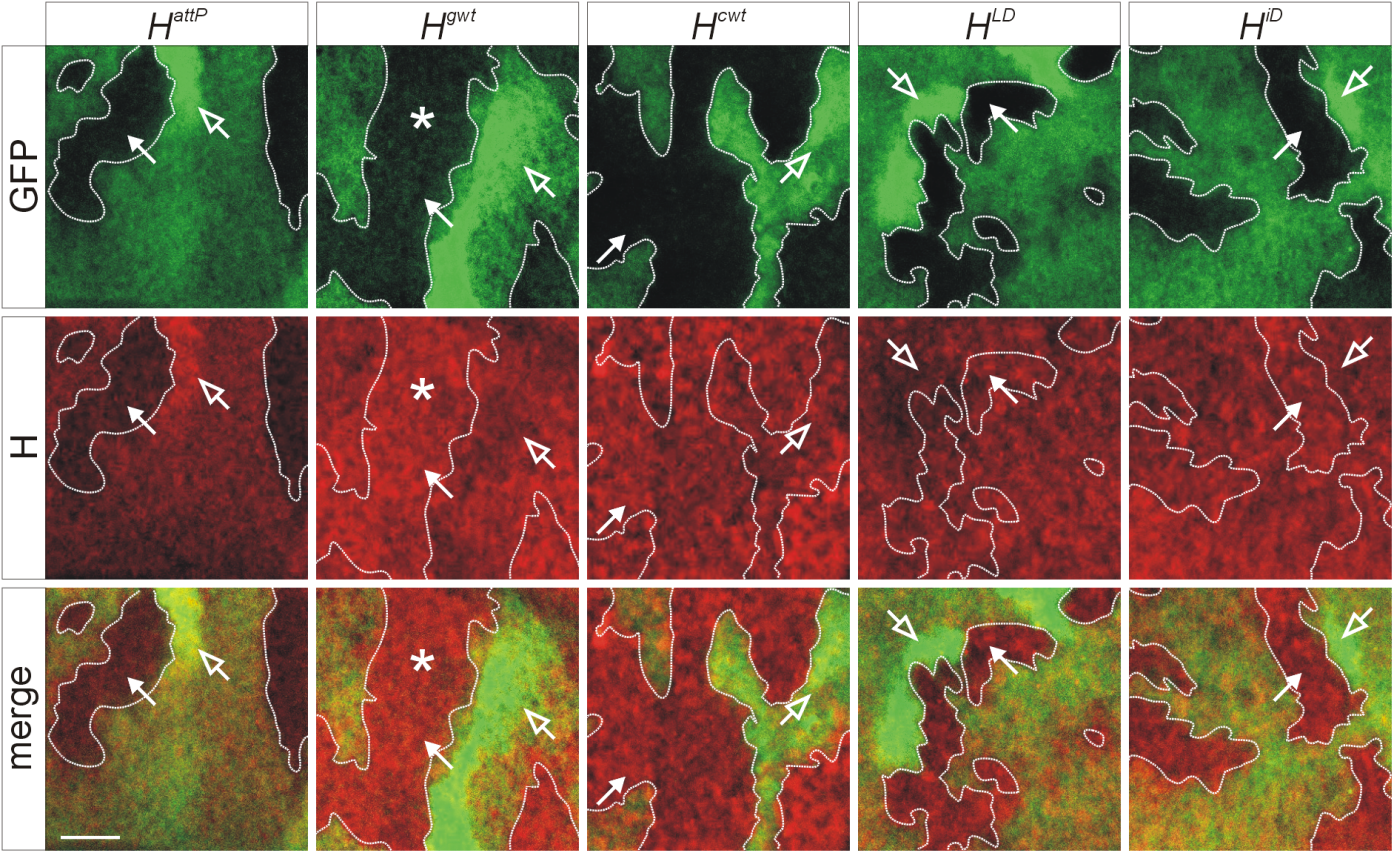
Protein expression in the new *H* alleles. Homozygous cell clones of the given allele were induced in wing imaginal discs in heterozygous larvae; they are unmarked and outlined with a dotted line for clarity. The heterozygous and the homozygous wild type cells are marked with GFP (lightly and strongly labelled in green, respectively). H protein is shown in red. Closed arrows point to an example of a homozygous *H* cell clone, and open arrows to an example of a homozygous wild type cell clone. Note near absence of H protein in the *H* ^*attP*^ homozygous cells and the slight enrichment of H protein in homozygous *H* ^*gwt*^ cells (asterisk), whereas *H* ^*cwt*^, *H* ^*LD*^and *H* ^*iD*^ cells produce H protein similar to wild type. Size bar corresponds to 20 μm.

Both *H* ^*LD*^ and *H* ^*iD*^ were designed as single amino acid substitutions, which are expected to produce normal amounts of H proteins, whereas *H* ^*attP*^ is expected to be protein null. H protein expression is notoriously difficult to detect on Western blots due to the low amount of expression [31]. We therefore performed a clonal analysis of the respective alleles using the Flp/FRT technique [32], allowing a comparison of H protein expression levels in the homozygous mutant cells with those of the heterozygous or homozygous wild type cells (Fig 5). As expected, H protein was nearly absent in the *H* ^*attP*^ clones–residual staining may result from background or from leftover parental protein (Fig 5). In contrast, H protein was well expressed in cell clones homozygous for either *H* ^*gwt*^ or *H* ^*cwt*^ and likewise for either *H* ^*LD*^ or *H* ^*iD*^ at levels matching those of the heterozygous or of the wild type sibling cells (Fig 5). Unexpectedly, H protein appeared enriched in cells homozygous for *H* ^*gwt*^ (Fig 5), suggesting a stronger expression or stability of the protein despite the apparent subtle reduction in activity (Fig 3 and S2 Fig).

**Fig 6:**
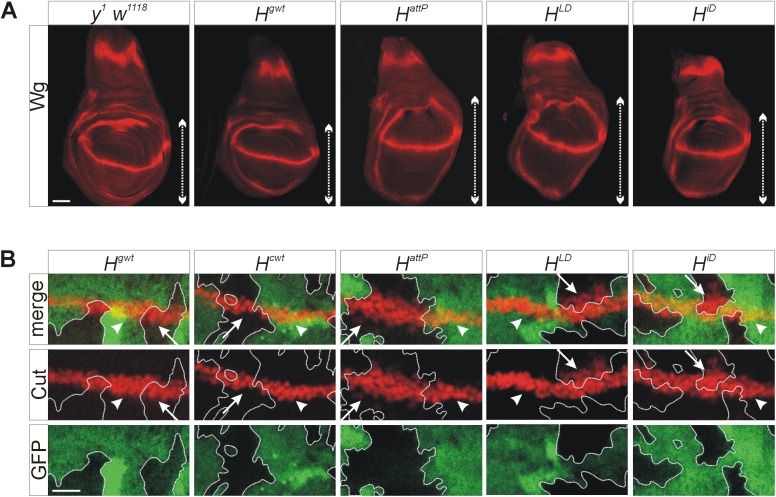
Notch gain of function phenotypes in the new *H* alleles. (A) Wing imaginal discs of homozygous larvae of the given genotypes were assayed for Wingless (Wg) expression (red). Wing blade size is marked by a double headed arrow; note overgrowth in *H* ^*attP*^, *H* ^*LD*^ and *H* ^*iD*^ mutants, whereas *H* ^*gwt*^ appears similar to the *y* ^*1*^ *w* ^*1118*^ control. Size bar corresponds to 50 μm. (B) Homozygous cell clones of the respective *H* allele as indicated were induced in wing imaginal discs stained for Cut protein (red), which is expressed along the dorso-ventral boundary. Wild type cells carry two copies of GFP and are strongly labelled green, whereas heterozygous cells with one copy are lightly labelled. The mutant cell clones are unlabelled (black) and outlined for clarity. Arrows point to Cut expression in the homozygous mutant cells, arrowheads in the control cells. Size bar corresponds to 20 μm.

Loss of the Notch antagonist H results in a gain of Notch activity, effecting for example a size increase in the wing imaginal discs [20,33,34], which we observed in *H* ^*attP*^ as well as in *H* ^*LD*^ or *H* ^*iD*^ homozygous mutant animals, whereas *H* ^*gwt*^ wing discs appeared wild type (Fig 6A). In addition Wingless protein expression, which is under the control of Notch signalling activity along the dorso-ventral boundary of the wing anlagen [34–36], was monitored. As expected, the Wingless stripe appeared slightly broader in the mutants compared to the rescue flies or the control (Fig 6A).

We next analysed the expression of Cut protein: normally Cut accumulates in a 2–3 cells wide strip along the dorso-ventral boundary of the wing imaginal disc in response to Notch signalling activity [20,37,38] (Fig 6B). To this end, homozygous mutant cell clones were generated and stained for Cut protein. Whereas Cut protein expression was undisturbed in homozygous *H* ^*gwt*^ or *H* ^*cwt*^ cells, the stripe appeared broader in homozygous mutant *H* ^*attP*^ cell clones touching the dorso-ventral boundary (Fig 6B). A similar broadening of Cut expression was seen in cells homozygous for either *H* ^*LD*^ or *H* ^*iD*^, demonstrating lack of H repressor activity in the mutants despite normal H protein expression (Figs 5 and 6B).

### Phenotypic classification of the *Hairless* mutants

The *H* ^*2*^ allele has been often used as a reference in the past [25,39–41]. *H* ^*2*^ has been described as an amorphic allele with regard to bristle loss and wing venation phenotype [25,41] or as a weak allele with regard to lethality in trans over other *H* alleles [39]. To address pupal lethality, the mutant alleles were crossed *inter se*, to the null allele *H* ^*attP*^ as well as to *H* ^*2*^and *H* ^*P8*^ [39], and the weak hypomorphic *H* ^*22*^ allele [25], included as a reference. Pupae were counted in relation to their TM6B siblings; homozygotes were rare and not recorded (Table 1). As observed before, the *H* ^*P8*^ allele was strongest with regard to lethality giving only a quarter of the expected *H* ^*attP*^/*H* ^*P8*^ trans-heterozygotes, whereas nearly 90% of the expected *H* ^*attP*^/*H* ^*2*^ pupae appeared. *H* ^*22*^ combinations even outnumbered the TM6B siblings, however, unexpectedly no *H* ^*22*^ homozygotes were obtained in our culture [25]. Both, *H* ^*LD*^ and notably *H* ^*iD*^ gave higher numbers of trans-heterozygotes than *H* ^*attP*^ in this assay, suggestive of residual H activity (Table 1).

**Table 1:** Survival of trans-heterozygous *H* mutants.

	*H* ^*P8*^	*H* ^*2*^	*H* ^*attP*^	*H* ^*LD*^	*H* ^*iD*^	*H* ^*iD*^
*H* ^*P8*^	-	92.5% (1047)	24.2% (1140)	36.1% (1595)	39.3% (1370)	105.1% (1344)
*H* ^*2*^		-	88.6% (977)	84.0% (1163)	90.6% (1046)	122.3% (1099)
*H* ^*attP*^			-	28.5% (1714)	53.1% (1401)	114.2% (1400)
*H* ^*LD*^				-	63.8% (1518)	122.9% (1665)
*H* ^*iD*^					-	127.9% (1577)

Shown is percentage of expected pupae as determined from the number of heterozygous siblings, balanced over TM6B *Tb*. Total number is given in parentheses.—Homozygotes were not recorded.

As second criterion for a classification of the new *H* alleles, the loss of macrochaetae was recorded in the heterozygous mutants (S3 Fig). As described before this phenotype is highly dependent on genetic background [41], perhaps explaining the differences between *H* ^*2*^ and *H* ^*attP*^ (S3 Fig). However, *H* ^*LD*^ and *H* ^*iD*^ alleles have formally the same genetic background; both derived from *H* ^*attP*^. Again, both *H* ^*LD*^ and *H* ^*iD*^ appeared somewhat weaker compared to *H* ^*attP*^ or *H* ^*2*^, with *H* ^*LD*^ being more similar to the two references (S3 Fig). Finally, all alleles were crossed with the hypomorphic allele *H* ^*22*^ [25]. Trans-heterozygotes were viable lacking all macrochaetae and many microchaetae on head and thorax (Fig 4C). Overall, these analyses allow arranging the alleles into the following phenotypic series: OreR ≈ *H* ^*gwt*^ > *H* ^*cwt*^ >> *H* ^*22*^ >> *H* ^*iD*^ > *H* ^*LD*^ > *H* ^*attP*^.

### Genetic interactions of new *Hairless* alleles with *Notch* and *Delta* mutants

**Fig 7:**
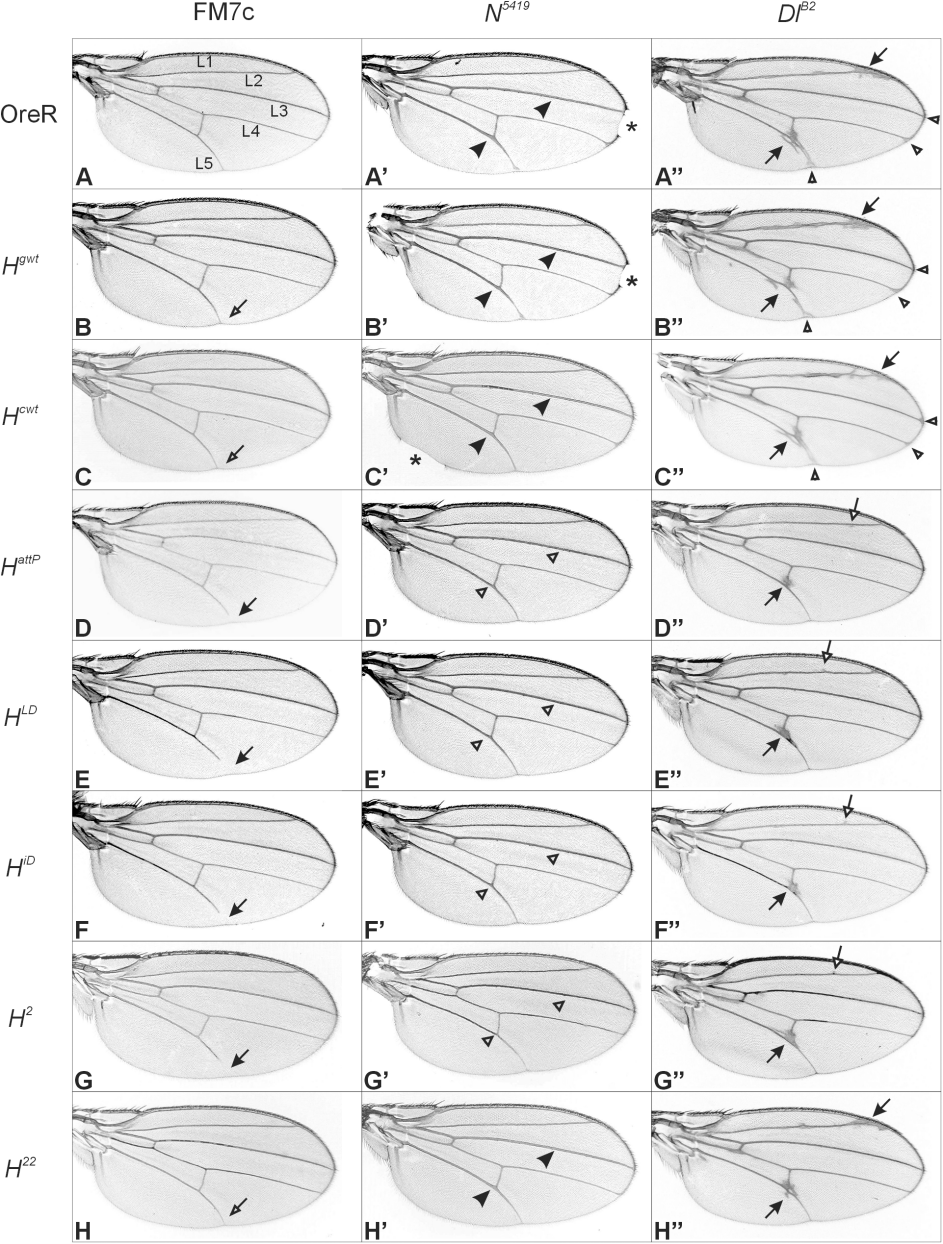
Genetic interactions of *H* alleles with *Notch* and *Delta* mutants. (A) Wild type control wing (FM7c in OreR); longitudinal veins L1—L5 are indicated. (B-H) The given *H* allele is heterozygous over wild type in the background of the FM7c balancer chromosome. Open arrows point to the distal end of L5 which is typically shortened in *H* mutants (closed arrow). (A’) Wing of a heterozygous *N* ^*5419*^ mutant in OreR wild type background. (B’-H’) Wings of trans-heterozygotes *N* ^*5419*^/+ *H*/+ (+ corresponds to the respective wild type Oregon R chromosome, *H* allele as indicated). Typical of *N* mutants are wing margin incisions (asterisks) and thickened L3 and L5 longitudinal veins that are marked by arrowheads. Loss of H activity results in vein thinning (open arrowheads). (A”) Wing of a heterozygous *Dl* ^*B2*^ mutant, characterized by ectopic vein material notably along the L2 and L5 veins (arrows), as well as distal deltas (open arrowheads). (B”-H”) Trans-heterozygous *Dl* ^*B2*^/*H* mutants. Ectopic veins are mostly present at the intersection of the L5 and the posterior cross-vein (arrow), plus some tiny veinlets along the L2 (open arrow).

Genetic interactions between *H* and Notch-pathway mutants have been amply described [13,19,39,42,43]. Heterozygous mutants of either the *Notch (N)*, *Delta (Dl)* or *H* locus display dominant wing phenotypes, i.e. wing margin incisions and thickened wing veins, which were name giving for the *N* and *Dl* gene, respectively, and shortened longitudinal wing veins for the *H* gene [25,39,40,42,44] (Fig 7). It has been observed before that a loss of one *H* gene copy ameliorates or even abrogates either *N* or *Dl* mutant wing phenotypes and vice versa [39,42,45]. In our culture conditions, about 91% of the amorphic *N* ^*5419*^ allele displayed notching of one or both wings (Fig 7A and 7A’ and S4 Fig). Combined with each *H* loss of function allele tested here, wing notching was very rare (Fig 7D–7H and 7D’–7H’ and S4 Fig). Whereas the *H* ^*gwt*^ allele was largely indistinguishable from the wild type Oregon R control, we observed a lowered frequency of notches in *N* ^*5419*^/+; *H* ^*cwt*^/+ trans-heterozygotes, supporting the earlier observation of a subtle loss of H activity in this allele (Fig 7A–7C and 7A’–7C’ and S4 Fig). At the same time, the other typical phenotype of heterozygous *N* mutant wings, namely thickening of L3 and L5 veins, was likewise rescued by a mutation in *H*, no matter which allele was used, but not by the rescue alleles (Fig 7A–7H’). Moreover, the shortening of the L5 vein typical of *H* mutants (Fig 7D–7G), disappeared in the trans-heterozygous combinations as well (Fig 7D’–7G’).

Heterozygous *Dl* mutants display thickened and knotted veins that broaden distally to form deltas at the wing margin [40,42], exemplified in Fig 7A” for the amorphic allele *Dl* ^*B2*^. It has been shown before that the *Dl/H* trans-heterozygotes display a more normal wing venation pattern [42]. Rescue of the *Dl* wing phenotype was indeed observed in the trans-heterozygous combination of *Dl* ^*B2*^ with any of the tested *H* alleles, except for the rescue constructs *H* ^*cwt*^ or *H* ^*gwt*^ as was expected (Fig 7A”–7H”). Quantitative evaluation of the differences confirmed the impression given by the pictures and the phenotypic series established above (S4 Fig). These data demonstrate that the engineered mutants *H* ^*attP*^, *H* ^*LD*^ and *H* ^*iD*^ are indeed loss of function *H* alleles, albeit *H* ^*LD*^ and notably *H* ^*iD*^ apparently retain some H activity and may hence be classified as strong hypomorphic mutants.

## Discussion

In this work we have applied the method of genomic engineering to generate new alleles in the *H* locus. Genomic engineering permits defined genetic modifications at the locus of choice to eventually study its regulation or function. The directed replacement of the locus with any desired allele is straightforward once the attP-founder line is established. The newly arising Crispr/Cas9 system is going to speed up this approach for the simplicity to replace a given locus for example with an attP landing site [46,47]. We have shown that the *H* ^*attP*^ founder line can be used to generate new wild type or mutant *H* alleles. Alternatively, *H* could be replaced by other known inhibitors of the Notch pathway to address their repressive potential during fly development, be it fly or mammalian components [11,48]. It will be interesting to see for example, whether mammalian Notch-repressors like KyoT2, whose structural interaction with the mammalian Su(H) homologue is known [49], are able to replace H function in *Drosophila*.

The *H* ^*attP*^ founder line deletes the entire *H* coding region and may serve henceforth as a reference for a *H* null allele. Both *H* ^*LD*^ and *H* ^*iD*^ mutants retain some *H* activity compared to *H* ^*attP*^, albeit the differences are subtle. The fact that *H* ^*iD*^ appears weakest suggests some residual Su(H) binding of this mutant, which however could not be detected in the yeast two-hybrid experiments. We have to keep in mind that both mutations were generated in the *H* cDNA, which in its wild type form is not sufficient to provide *H* activity completely. Unfortunately, we cannot distinguish whether the *H* mutant phenotype in the *H* ^*LD*^ and *H* ^*iD*^ alleles is aggravated by the incomplete activity of the cDNA construct and if so, to what degree. It is conceivable that either point mutation, although strongly affecting the binding of Su(H), only partly affects *H* activity.

Assuming that H^LD^ and H^iD^ fail to bind to Su(H), the residual activity of these alleles implies additional, Su(H)-independent activities of H. This is, however, not unexpected since H has been shown by genetic and molecular means to interact with other factors but Su(H), apart from the binding to its corepressors Groucho and CtBP [7,9,10,12,14,16–18,29,42,50–54]. For example, the binding of Pros26.4 to H results in a destabilization of H protein, thereby promoting Notch signalling activity independent of Su(H) (Müller et al. 2006). More recently it was shown that H interacts with Runt thereby participating in Runt-dependent transcriptional regulation of segmentation genes during *Drosophila* embryogenesis [52]. A complete loss of *H* is hence expected to affect fly development more profoundly than just the lack of Su(H) binding, i.e. repression of Notch signalling activity.

It came to our surprise that the *H* cDNA was not able to fully rescue the *H* mutant phenotypes. The *H* gene is expressed at low to moderate level in all tissues throughout development with some enrichment in the larval brain (http://www.flybase.org; http://www.flyatlas.org) [39,55]. Accordingly, a *H* cDNA under heat shock promoter control was able to rescue the haplo-insufficient dominant phenotype of *H* heterozygotes even at ambient temperatures (Bang and Posakony 1992), suggesting that a moderate *H* expression may suffice for full *H* activity. Our analysis does not support this view; instead it favours the presence of as yet unidentified regulatory elements within the *H* introns. In search of such regulatory elements we compared *H* intron sequences within the genus *Drosophila*, comprising species of the Sophophora and Drosophila subgenera that have been split about 40 million years ago (http://www.flybase.org/blast). This distance is large enough to detect regulatory elements on ground of their conservation as exemplified for the *fushi tarazu* gene [56,57]. No conservation outside of the subgenus Sophophora was however observed suggesting a lack of intronic regulatory elements. Maybe the elements are too small to be detected this way. Likewise, the sequences in the *H* 5’ or 3’ UTR that were altered in the course of genomic engineering, are not conserved between *D*. *melanogaster* and other *Drosophila* species except for sibling species within the melanogaster subgroup. This leaves us with no simple explanation for the restricted activity of the *H* cDNA construct. Perhaps the manipulation of the genome has resulted in a sensitized genetic background uncovered in the hemizygous condition.

Both *H* ^*cwt*^ and *H* ^*gwt*^ homozygotes are fully viable without apparent phenotype, which demonstrates the functionality of the constructs. Notably, the reduced activity is only observed in the hemizygous condition as an enhancement of the *H* haplo-insufficient phenotype. Most other fly genes are fully recessive, i.e. normally such a subtle decrease in activity may go unnoticed. *H* heterozygotes in contrast appear to provide a highly sensitized genetic background regarding bristle development. This has been observed decades ago during systematic analyses of the *H* loss of bristles phenotype, which was shown to be highly background dependent [41]. In fact, three copies of *H* result in gain of function phenotypes in support of the notion that *H* activity is strictly dose sensitive [39,42,58]. Accordingly, *H* belongs to the small number of known quantitative trait loci implicated in natural variation of bristle number [59,60]. Quantitative traits are generally sensitive to the environment, effected by the simultaneous segregation of alleles at multiple loci. This explains easily the strong influence of the genetic background on the *H* bristle loss phenotype.

Against expectations we observed an enrichment of H protein in homozygous *H* ^*gwt*^ cells compared to cells bearing the GFP-marked third chromosome used in the FRT-mediated mosaic analysis in one or two copies (Fig 5). In fact the *H* locus shows extraordinary fluctuations of transcriptional activity within but not between species [61]: in this work 10 inbred *D*. *simulans* strains were compared with a pool of isogenic *D*. *melanogaster* strains. Perhaps, our mosaic analysis visualized these results in a direct way, i.e. differential levels of *H* expression in different genetic backgrounds. Usually gene expression is remarkably precise and reproducible within cell populations, despite the random molecular fluctuations expected to occur between individual cells. It has been proposed that this transcriptional noise is filtered through the coordinated activity of the two signalling pathways Wnt and Notch [62,62,63]. Hairless, as the major antagonist of Notch signalling activity in *Drosophila*, may be at the heart of this regulation, since its activity has a direct influence on Notch signalling output [11]. Perhaps the fluctuation in the expression of *H* uncovers the regulatory mechanisms implemented in filtering transcriptional noise.

## Materials and Methods

### Genome engineering methods

Genome engineering was performed as outlined before [23]. At first a 3 kb *Bam* HI/*Kpn* I 5’ flanking fragment (5’arm) and a 6.4 kb *Eco* RV/*Xho* I 3’ flanking fragment (3’arm) were excised from genomic subclones [39], and cloned into the 5’ and 3’ MCS of pGX-attP. Five independent transgenic pGX-H fly lines were established by random P-element mediated germ line transformation. To induce homologous recombination, a second chromosomal insertion line was chosen and crossed with *y* ^*1*^ *w* ^*1118*^; P{ry*, 70Flp}11P{*v* ^*+*^,70I-SceI}2B *Sco*/CyO (BL6934) under a heat shock regimen as outlined before [24]. One recombination event *H* ^*attP w+*^ was obtained from roughly 1000 virgins bearing mosaic eye colour, crossed with w*; P{70FLP}10 (BL6938). It was confirmed by phenotype and PCR analysis using the primer pair P1 (upstream of 5’ arm) and P2 (within attP). Vector sequences including the *white* ^+^ marker gene were deleted by CreI-mediated recombinase using *y* ^*1*^ *w* ^*67c23*^ P{Crey}1b; D*/TM3 *Sb* (BL851) as described before [24]. The resultant *H* ^*attP*^ mutant was stabilized over balancer chromosomes and confirmed by PCR using primer pair P3 and P4. In addition, the 1.3 kb amplificate was subcloned and sequence verified.

Generation of the *H* integration constructs followed largely the same strategy: all constructs cover the *Kpn* I/*Eco* RV fragment deleted in *H* ^*attP*^ (position 415 to 4187 according to FlyBase). The H-cWT construct contains the entire *H* cDNA (h9/h7) [39], starting with the *Kpn* I site in the 5’ UTR and ending at position 4323 within the 3’ UTR [14,39], i.e. 136 nt downstream of the *Eco* RV site (S1 Fig). At first the MCS of the original pBT H cDNA subclone [14] was extended by insertion of a *Kpn* I site between the 3’ flanking *Xba* I and *Sac* I site using annealed primers. This way, the entire 4.2 kb insert could be excised as one *Kpn* I fragment and inserted into likewise opened pGE-attB^GMR^ vector [23]. The same strategy was used for H-LD and H-iD cDNAs. Orientation was tested by confirmative restriction digests. Moreover, the H-LD and H-iD replacements were confirmed by diagnostic digests.

The genomic 5.1 kb H-gWT construct spans from *Kpn* I to *Eco* RV containing all the introns. It was gained from the HBS genomic subclone [39], which however, lacks the 5’ *Kpn* I site due to a polymorphism (genotype *Kr* ^*SB10*^/SM1). Hence, we replaced an overlapping 1.9 kb *Eco* RI fragment with one containing the *Kpn* I site, derived from a different genomic library (H-clones, genotype Oregon R; [39]). Shuttling into pGE-attB^GMR^ vector was as described above using the flanking *Kpn* I sites. Fly stocks were confirmed by PCR before and after floxing using primer pairs P3/P5 and P6/P4, respectively (S1 Fig). The genotypes of *H* ^*gwt*^ and *H* ^*cwt*^ were further confirmed by sequencing the junctions of a P7-P4 amplificate with primers S8 and S9. Primers are listed in S1 Table.

Insertion of DNA constructs into the *H* ^*attP*^ genome was performed exactly as outlined before [23]. To this end, about 500–1000 embryos derived from a cross of w*; *H* ^*attP*^/TM6B males with *y* ^*1*^ M {vas-int.Dm}ZH-2A w* (BL40161) females expressing the phiC31 integrase [28], were injected and transgenic lines established. The frequency of recombinants ranged between 0.7%–23.3% of first generation crosses. The pGE-attB^GMR^ and *white* ^+^ marker sequences were eliminated with Cre-recombinase by crossing in *y* ^*1*^ *w* ^*67c23*^ P{Crey}1b; D*/TM3 *Sb* (BL851). White eyed stocks lacking the recombinase and balanced over TM6B were established for each construct. The correct genotype was confirmed by PCR analyses and sequencing.

### Yeast two-hybrid experiments

Yeast two-hybrid experiments were performed according to standard protocols [64,65], using pJG Su(H) [10] as prey and pEG NTCT [30], pEG NTCT-L235D [15] or pEG NTCT-I244D (this work) as bait. Empty vectors served as control. QuikChange^®^ II XL Site-Directed Mutagenesis Kit (Agilent Technologies, Böblingen, Germany) was used to introduce the I244D exchange in NTCT [30] according to the manufacturer’s protocol. Primers iDup and iDlo are listed in S1 Table. The clone was sequence verified and was shuttled as *Bam* HI/*Xho* I fragment into likewise opened pEG202 vector [66] for yeast two-hybrid experiments.

### Fly work

Fly husbandry was on standard fly food at 18°C; crosses were kept at 25°C. The following stocks were used: Oregon R, *y* ^*1*^ *w* ^*1118*^, Df(1)N–5419/FM7c [67], *Dl* ^*B2*^/TM6C *Sb* [37], *H* ^*2*^/TM6B [40], *H* ^*22*^/TM6B [25], *H* ^*P8*^/TM6B [31]. Number of trans-heterozygous *H* pupae were recorded taking advantage of the *Tubby* marker on the TM6B balancer chromosome. To this end, 15 virgin females were crossed with 8 males in eight parallel crosses, and the F1 examined for the *Tubby* marker. Numbers of macrochaetae were recorded exactly as described before on 20 individuals each [25].

Adult wings of female flies were dehydrated in ethanol and mounted in Euparal (Roth, Karlsruhe, Germany), dried over night, and pictures taken on a Zeiss Axiophot using an ES120 camera (Optronics, Goleta CA, USA) and Pixera Viewfinder software, version 2.0. Pictures of uncoated, etherized adult flies or unshelled pharate adults were captured with a table-top scanning electron microscope (Neoscope JCM–5000; Nikon, Tokyo, Japan). Significance was determined by one tailed Student’s T-test. Not significant, ns p>0.05; significant * p<0.05, ** p<0.01, *** p<0.001 (http://www.physics.csbsju.edu/stats/t-test_bulk_form.html).

Clones were induced using the Flp/FRT technique as outlined before [17,32,68] using either *H* ^*gwt*^, *H* ^*cwt*^, *H* ^*attP*^, *H* ^*iD*^ or *H* ^*LD*^ allele recombined with P{neo FRT}82B (BL2050) and crossed with P{neo FRT}82B P{Ubi-GFP^S65T^nls}3R/TM6B (BL32655). FLPase was induced by a 1h heat shock at 37°C in first to second instar larvae, to be dissected as wandering third instar.

### Immuno-staining of wing imaginal discs

Immuno-staining of imaginal discs was according to standard protocols using Hairless anti-A (1:500, from guinea pig) [21], mouse anti-Cut (1:25) or anti-Wingless (1:25) (developed by G. Rubin and S.M. Cohen, respectively; obtained from the Developmental Studies Hybridoma Bank developed under the auspices of the NICHD and maintained by the University of Iowa, Dept of Biology, Iowa City, IA 52242) and rabbit anti-GFP (1:200, Santa Cruz Biotech, Dallas, USA). Goat secondary antibodies coupled to FITC, Cy3 or Cy5 were from Jackson Immuno-Research (Dianova, Hamburg, Germany). Tissue was mounted in Vectashield (Vector Labs, Eching, Germany), and examined using a BioRad MRC1024 confocal microscope and LaserSharp 2000TM software (Carl Zeiss, Jena, Germany).

## Supporting Information

S1 Fig Reintegration of *H* variants. Schematic representation of the integration events at the *H* locus (not to scale). A) The pGE-attB^GMR^ vector, containing the *H* construct of interest (H*) and a *white* ^+^ marker, is recombined via its attB site into the attP site present in place of the *H* locus in the *H* ^*attP*^ allele. B) As an example, the resulting flies carry the entire vector at the *H* locus, which was confirmed by a 5’H and a 3’H PCR (see D). Due to the recombination event, attP and attB have been changed to attR and attL. C) Vector sequences and the *white* ^+^ marker were removed by Cre-mediated recombination at the loxP sites. In the end the *H* gene is replaced by the given allele; only the attR and the loxP sites remain in the 5’ and the 3’UTR, respectively. D) Control PCR reactions performed on single flies before floxing (see B). M, marker (appr. size in kb); 1, *H* ^*attP w+*^/TM6B; 2, *H* ^*cw w+t*^; 3, *H* ^*LD w+*^/TM6B; 4 *H* ^*iD w+*^/TM6B, 5, *H* ^*gwt w+*^. For the 5’H PCR a 2.28 kb is expected for the wild type chromosome (arrow); a 2.39 kb fragment after introduction of the genomic H-gwt construct (size increase due to attR); a 1.64 kb fragment after the introduction of *H* cDNA constructs lacking the introns (arrowhead) (primer pair P3/P5). Asterisk, unspecific priming. Using primer pair P6/P4, the 3’H PCR is expected to give a 1.25 kb fragment in case of a successful integration event (arrowhead). D’) Control PCR reactions performed on single flies after floxing (see C). The 5’H PCR is expected to give the same results as in D). With the 3’H PCR no amplificate is expected; as control unfloxed *H* ^*gwt w+*^ was included (arrowhead). Asterisk, unspecific priming. M, marker (appr. size in kb); 1, *H* ^*attP*^/TM6B; 2, *H* ^*cwt*^; 3, *H* ^*LD*^/TM6B; 4, *H* ^*iD*^/TM6B; 5, *H* ^*gwt*^; 6, *H* ^*gwt w+*^. E) Scheme of the *H* 3’ UTR to show position of changes relative to known or potential functional elements like polyadenylation sites [p(A)] and micro-RNA binding sites (miR). Target sites for several miRs are indicated as predicted by TargetScanFly (release 6.2; http://www.targetscan.org/fly_12). Purple, 8mer; red, 7mer-m8; blue, 7mer-1A). Numbering is according to FlyBase. Ends of major transcripts RA-RD (B/D, A/C), as well as of the cDNA NB15 [39] are indicated by an arrowhead. Polylinker sequences are shown in black for pBT and grey for pGX-attP; loxP is in yellow. The 136 nt sequence duplication, flanking the foreign sequences, is marked by an orange box; it does not contain known elements.
(TIF)

S2 Fig Statistical evaluation of bristle loss in hemizygous rescue genotypes. Statistical evaluation of bristle loss in homozygous *H* ^*cwt*^ vs. control *y* ^*1*^ *w* ^*1118*^ flies, and in hemizygous *H* ^*gwt*^ */H* ^*attP*^, *H* ^*cwt*^ */H* ^*attP*^ vs. +/*H* ^*attP*^ flies (+ corresponds to the third chromosome of *y* ^*1*^ *w* ^*1118*^), according to [25]. 40 macrochaetae were evaluated, 14 on the head, and 26 on the thorax. These are anterior, media and posterior Orbitals (Or), Ocellars (Oc), anterior and posterior Verticals (V) and Postverticals (PV) on the head; upper and lower Humerals (Hu), Presuturals (PS), anterior and posterior Notopleurals (NP), anterior and posterior Supra-Alars (SA), anterior and posterior Dorso-Centrals (DC), anterior and posterior Post-Alars (PA) and anterior and posterior Scutellars (Sc) (see scheme). 20 adult females were evaluated each. Error bars denote standard deviation. Statistical relevance was evaluated by a one tailed Student’s T-test (ns, not significant with p>0.05; * p<0.05; *** p<0.001).
(TIF)

S3 Fig Statistical evaluation of bristle loss in the new *H* alleles. Statistical evaluation of bristle loss in heterozygous *H* alleles as indicated; *y* ^*1*^ *w* ^*1118*^ flies served as control. For abbreviation see S2 Fig. 20 adult females were evaluated each. Error bars denote standard deviation. Statistical relevance was determined with a one tailed Student’s T-test (ns, not significant with p>0.05; * p<0.05; ** p<0.01; *** p<0.001).
(TIF)

S4 Fig Statistical evaluation of the genetic interactions with *Notch* and *Delta* mutants. A) Wild type (OreR) and the given *H* allele, respectively, were crossed with *N* ^*5419*^/FM7c virgins, and female offspring was evaluated for notched wings. 33–95 animals were analysed. B) Wild type (OreR) and the given *H* allele, respectively, were crossed with *Dl* ^*B2*^/TM6C *Sb* virgins. Wings of female offspring were evaluated for vein thickening at 13 positions indicated in the scheme. Thickening was recorded with a value of 1, no thickening with 0. Total number of analysed wings is given for each combination. Statistical relevance was determined with a one tailed Student’s T-test (ns, not significant with p>0.05; ** p<0.01; *** p<0.001; n, 17–28).
(TIF)

S1 Table List of primers, given in 5’ -> 3’. (DOC)
