# Supplementary figures and images for: Generation of New Hairless Alleles by Genomic Engineering at the Hairless Locus in Drosophila melanogaster
Source: PLoS One. 2015 Oct 8;10(10):e0140007. doi: 10.1371/journal.pone.0140007 (PMC4598140; doi:10.1371/journal.pone.0140007)

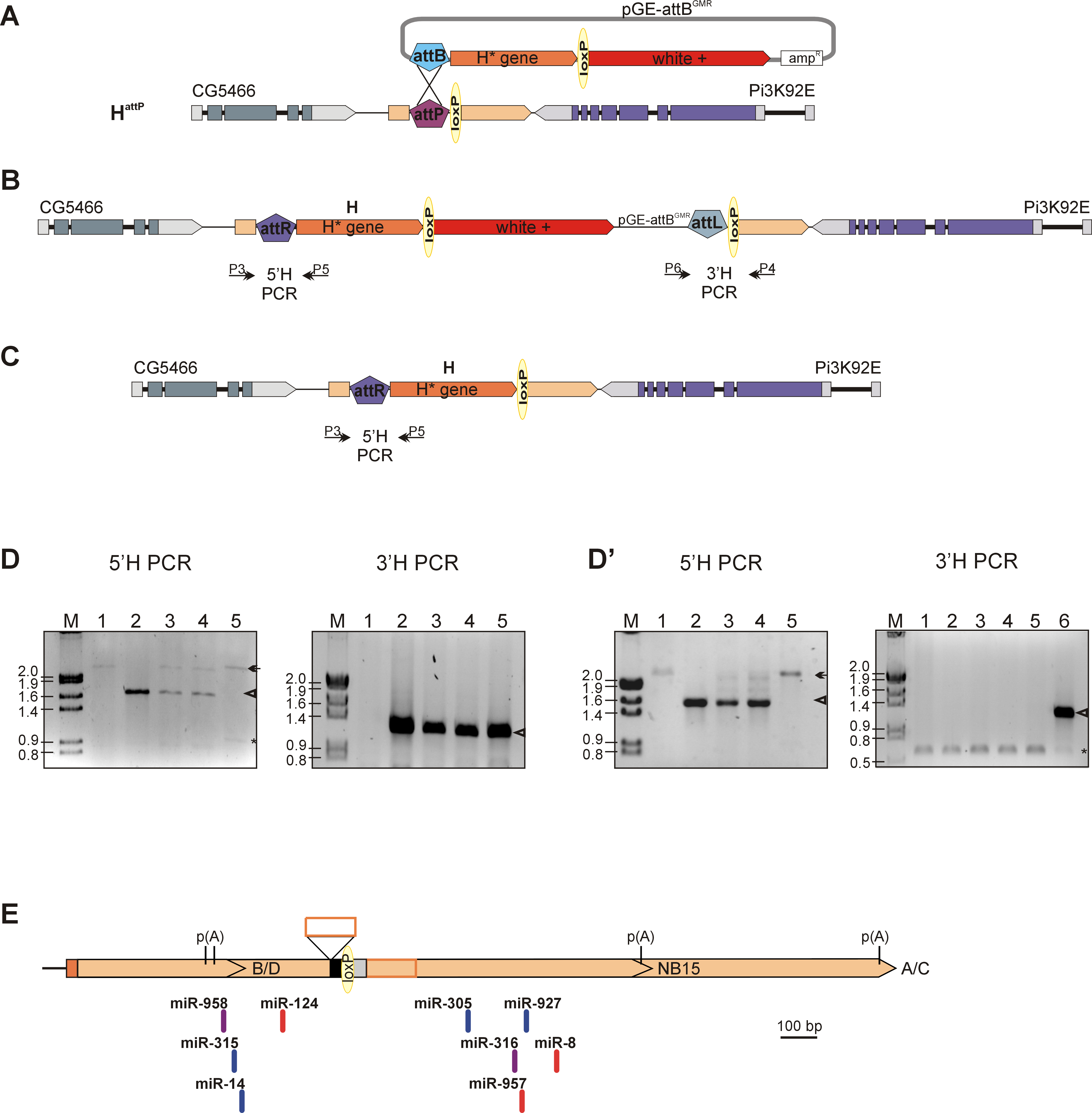

Supplement: S1 Fig — Schematic representation of the integration events at the H locus (not to scale). A) The pGE-attBGMR vector, containing the H construct of interest (H*) and a white + marker, is recombined via its attB site into the attP site present in place of the H locus in the H attP allele. B) As an example, the resulting flies carry the entire vector at the H locus, which was confirmed by a 5’H and a 3’H PCR (see D). Due to the recombination event, attP and attB have been changed to attR and attL. C) Vector sequences and the white + marker were removed by Cre-mediated recombination at the loxP sites. In the end the H gene is replaced by the given allele; only the attR and the loxP sites remain in the 5’ and the 3’UTR, respectively. D) Control PCR reactions performed on single flies before floxing (see B). M, marker (appr. size in kb); 1, H attP w+/TM6B; 2, H cw w+t; 3, H LD w+/TM6B; 4 H iD w+/TM6B, 5, H gwt w+. For the 5’H PCR a 2.28 kb is expected for the wild type chromosome (arrow); a 2.39 kb fragment after introduction of the genomic H-gwt construct (size increase due to attR); a 1.64 kb fragment after the introduction of H cDNA constructs lacking the introns (arrowhead) (primer pair P3/P5). Asterisk, unspecific priming. Using primer pair P6/P4, the 3’H PCR is expected to give a 1.25 kb fragment in case of a successful integration event (arrowhead). D’) Control PCR reactions performed on single flies after floxing (see C). The 5’H PCR is expected to give the same results as in D). With the 3’H PCR no amplificate is expected; as control unfloxed H gwt w+ was included (arrowhead). Asterisk, unspecific priming. M, marker (appr. size in kb); 1, H attP/TM6B; 2, H cwt; 3, H LD/TM6B; 4, H iD/TM6B; 5, H gwt; 6, H gwt w+. E) Scheme of the H 3’ UTR to show position of changes relative to known or potential functional elements like polyadenylation sites [p(A)] and micro-RNA binding sites (miR). Target sites for several miRs are indicated as predicted by TargetScanFly (release 6.2; ht [file pone.0140007.s001.tif]

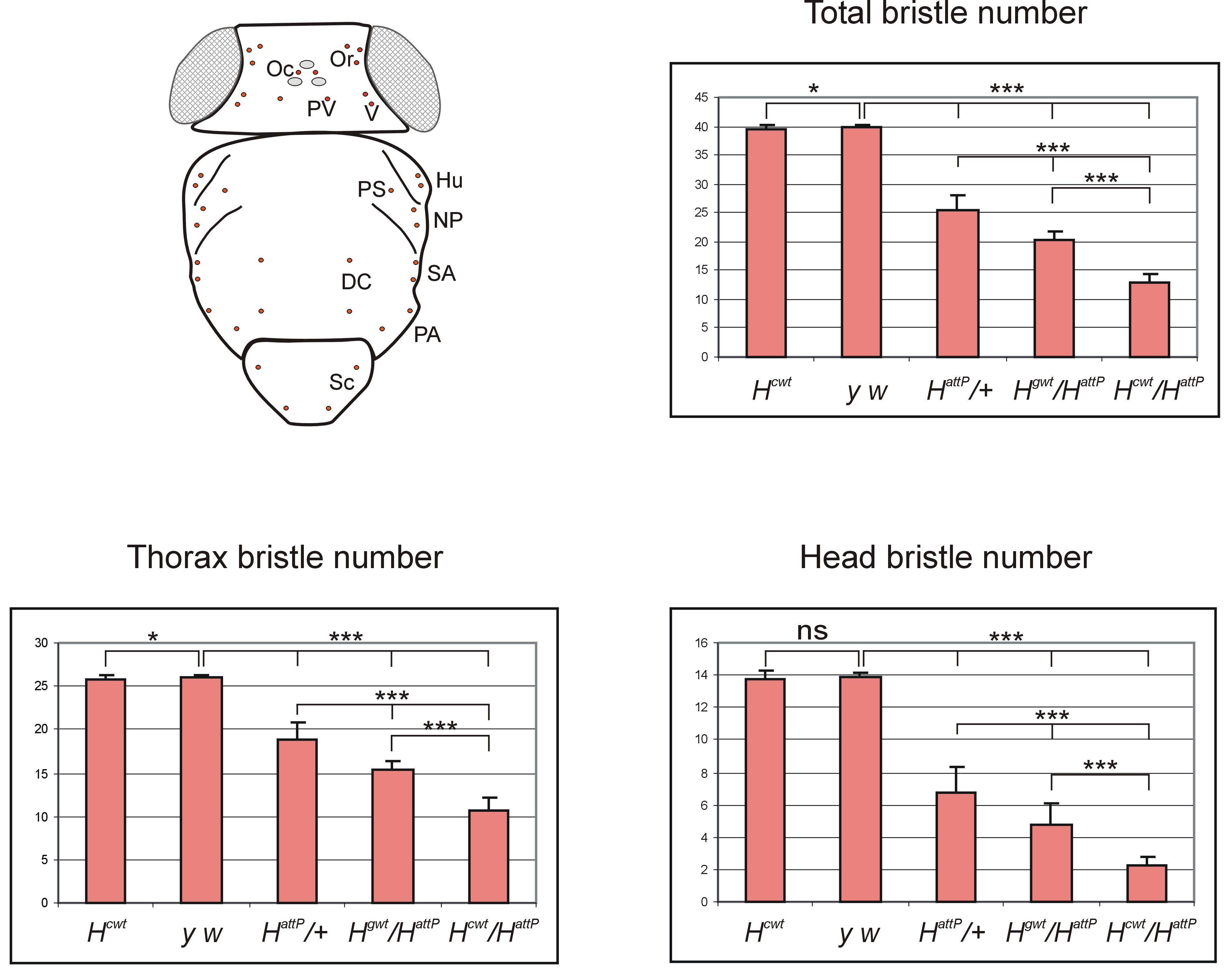

Supplement: S2 Fig — Statistical evaluation of bristle loss in homozygous H cwt vs. control y 1 w 1118 flies, and in hemizygous H gwt /H attP, H cwt /H attP vs. +/H attP flies (+ corresponds to the third chromosome of y 1 w 1118), according to [25]. 40 macrochaetae were evaluated, 14 on the head, and 26 on the thorax. These are anterior, media and posterior Orbitals (Or), Ocellars (Oc), anterior and posterior Verticals (V) and Postverticals (PV) on the head; upper and lower Humerals (Hu), Presuturals (PS), anterior and posterior Notopleurals (NP), anterior and posterior Supra-Alars (SA), anterior and posterior Dorso-Centrals (DC), anterior and posterior Post-Alars (PA) and anterior and posterior Scutellars (Sc) (see scheme). 20 adult females were evaluated each. Error bars denote standard deviation. Statistical relevance was evaluated by a one tailed Student’s T-test (ns, not significant with p>0.05; * p<0.05; *** p<0.001). (TIF) [file pone.0140007.s002.tif]

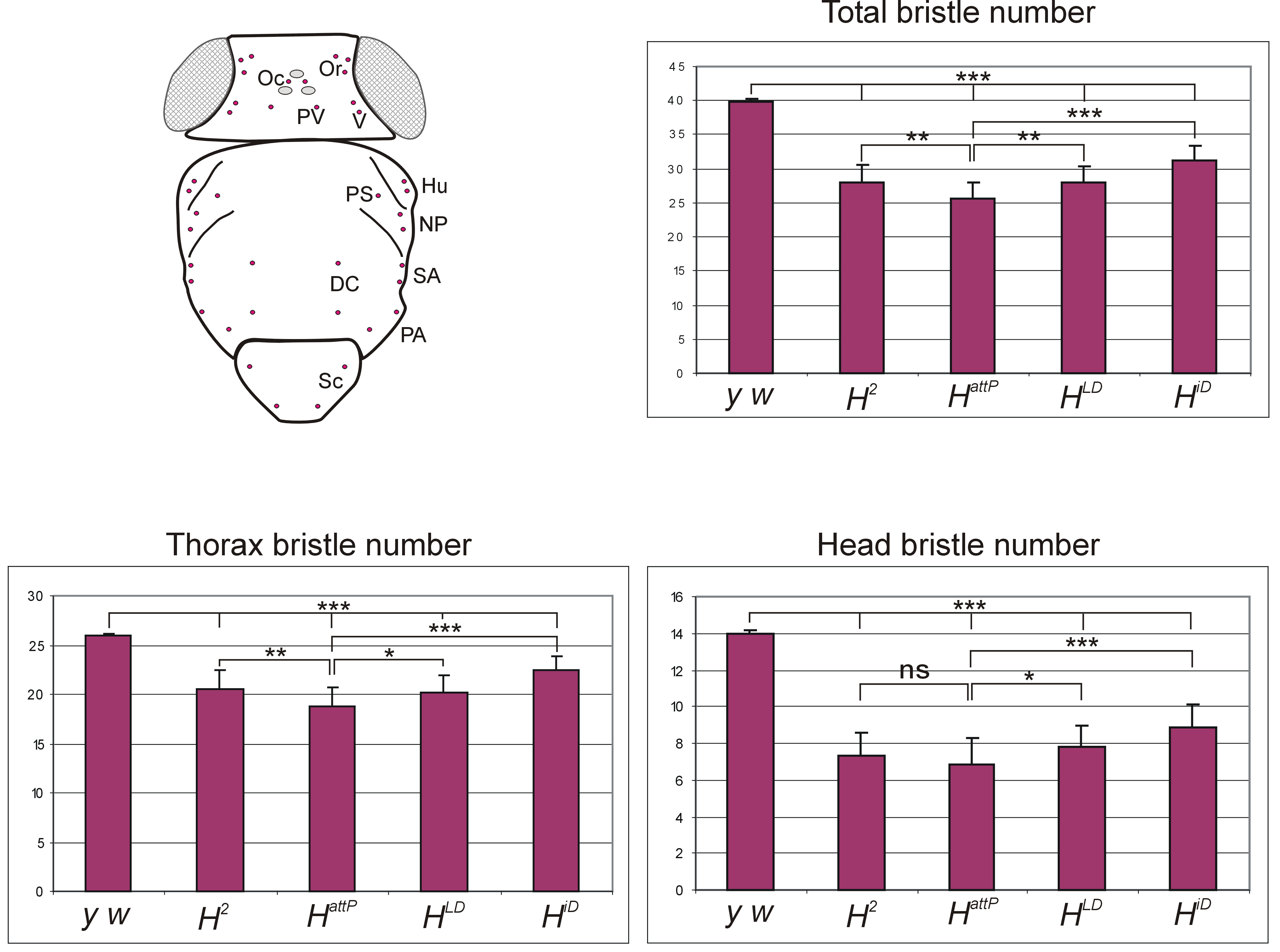

Supplement: S3 Fig — Statistical evaluation of bristle loss in heterozygous H alleles as indicated; y 1 w 1118 flies served as control. For abbreviation see S2 Fig. 20 adult females were evaluated each. Error bars denote standard deviation. Statistical relevance was determined with a one tailed Student’s T-test (ns, not significant with p>0.05; * p<0.05; ** p<0.01; *** p<0.001). (TIF) [file pone.0140007.s003.tif]

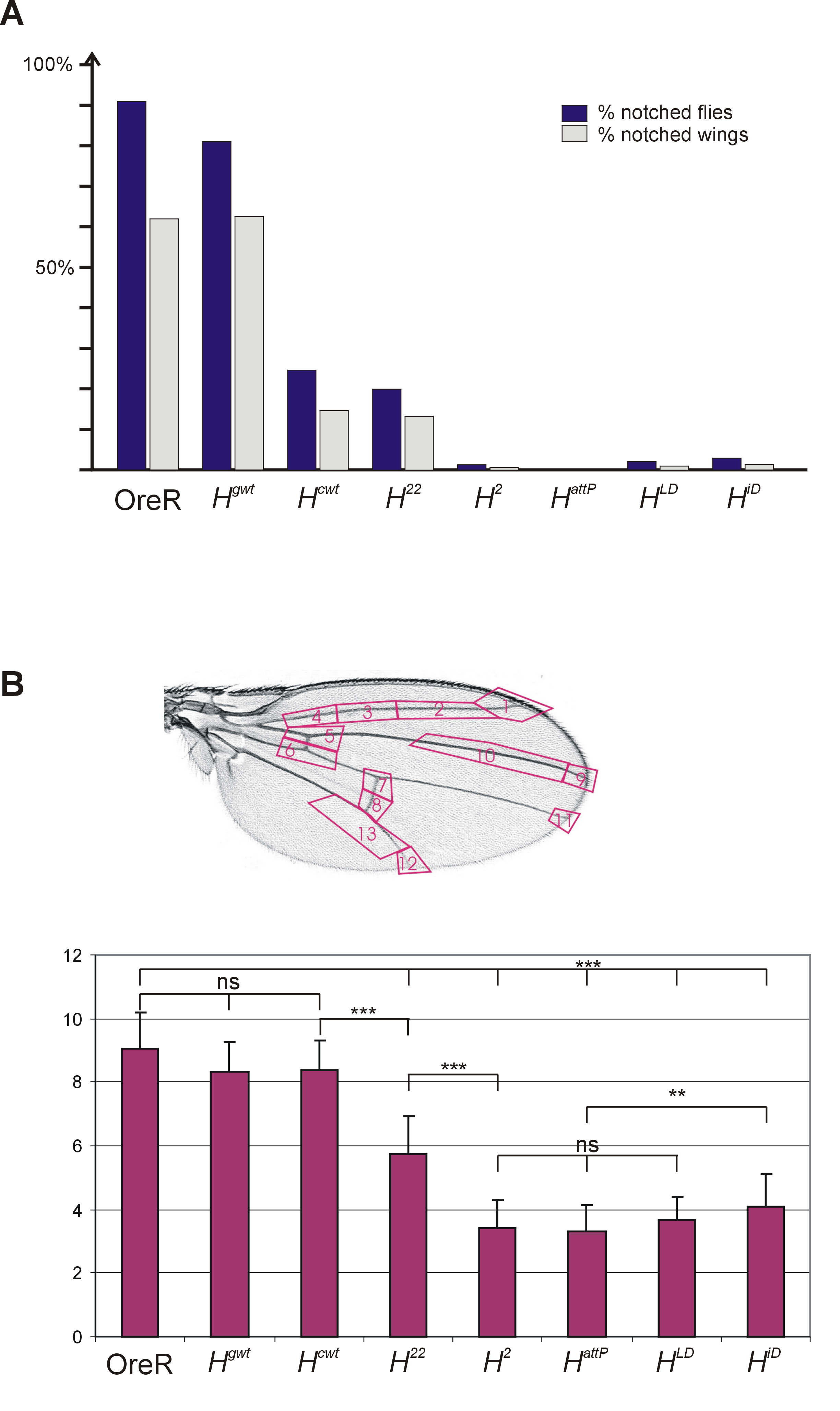

Supplement: S4 Fig — A) Wild type (OreR) and the given H allele, respectively, were crossed with N 5419/FM7c virgins, and female offspring was evaluated for notched wings. 33–95 animals were analysed. B) Wild type (OreR) and the given H allele, respectively, were crossed with Dl B2/TM6C Sb virgins. Wings of female offspring were evaluated for vein thickening at 13 positions indicated in the scheme. Thickening was recorded with a value of 1, no thickening with 0. Total number of analysed wings is given for each combination. Statistical relevance was determined with a one tailed Student’s T-test (ns, not significant with p>0.05; ** p<0.01; *** p<0.001; n, 17–28). (TIF) [file pone.0140007.s004.tif]
