# Supplementary material for: Generation of New Hairless Alleles by Genomic Engineering at the Hairless Locus in Drosophila melanogaster
Source: PLoS One. 2015 Oct 8;10(10):e0140007. doi: 10.1371/journal.pone.0140007 (PMC4598140; doi:10.1371/journal.pone.0140007)
Supplement: S1 Table — (DOC) [file pone.0140007.s005.doc]

S1 Table List of primers, given in 5’ -> 3’

| P1 | CGC CCA GCA AAA TTT AGA GAA GCT TAG CAA |
| --- | --- |
| P2 | CAC TAC GCC CCC AAC TGA GAG AAC TCA AAG |
| P3 | GAC TGG CGC CGT GGA TTT TGA CG |
| P4 | CAA GTT TCA GGC CGC CTT GTG CAA AC |
| P5 | GGG ACT GTT TCC AGG GCT GGT CTC G |
| P6 | CAT AAG GTG GTC CCG TCG GCA AGA GAC |
| P7 | CAT ACA TTC CCT CGC CTT CGA TAT |
| S8 | ATC AAA GCT TCC GCC GGA GTT G |
| S9 | TGG AGG TTA AAT TTA GAT CCG CCG |
| iDup | AGA TGG CAA ATT CGA TTT GGA GCT CGC GCG GTC C |
| iDlo | GGA CCG CGC GAG CTC CAA ATC GAA TTT GCC ATC T |
